# Hydrogen Protects Mitochondrial Function by Increasing the Expression of PGC‐1α and Ameliorating Myocardial Ischaemia–Reperfusion Injury

**DOI:** 10.1111/jcmm.70236

**Published:** 2024-11-27

**Authors:** Yue Zuo, Jiawei Wang, Zhexuan Gong, Yulong Wang, Qiang Wang, Xueyang Yang, Fulin Liu, Tongtong Liu

**Affiliations:** ^1^ Heart Center The First Hospital of Tsinghua University Beijing China; ^2^ School of Clinical Medicine Hebei University Baoding China; ^3^ Affiliated Hospital of Hebei University Baoding China

**Keywords:** H9C2rat myocardial cells, hydrogen, mitochondria, myocardial ischaemia–reperfusion injury, PGC‐1α

## Abstract

To investigate the application of H_2_ to alleviate cardiac ischaemia–reperfusion (I/R) injury in a PGC‐1α‐dependent manner. A rat in vitro myocardial I/R injury model was used, Western blot was used to detect the expression levels of apoptosis markers (Bax, cleaved caspase‐3, Bcl_2_), inflammatory factors (IL‐1β, TNF‐α), mitochondrial fission (DRP1, MFF) and mitochondrial fusion (MFN1, MFN2, OPA1). HE staining was used to observe the effect of H_2_ on the myocardial tissue structure injured by I/R. Transmission electron microscopy (TEM) was used to observe the changes in the mitochondrial structure of myocardial tissue after I/R injury. Real‐time quantitative PCR (qPCR) was used to detect the expression of PGC‐1α in the myocardial tissue of rats after I/R injury and H_2_ treatment. H_2_ increases the expression level of PGC‐1α, while the deletion of PGC‐1α inhibited the therapeutic effect of H_2_. H_2_ can improve the changes of the myocardial tissue and mitochondrial structure caused by I/R injury. H_2_ treatment effectively inhibited the inflammatory response, and the loss of PGC‐1α could inhibit the therapeutic effect of H_2_. The application of H_2_ can alleviate myocardial I/R injury, and the loss of PGC‐1α weakens the protective effect of H_2_ on the I/R heart.

**Abbreviations**
CABG: coronary artery bypass grafting
H_2_
: hydrogen
HR: hypoxia reoxygenation
MI: myocardial infarction
MIRI: myocardial ischaemia/reperfusion injury
mtDNA: mitochondrial DNA
OXPHOS: oxidative phosphorylation
PCI: percutaneous coronary intervention
PPARγ: peroxisome proliferator‐activated receptor γ

## 1 Introduction

Myocardial infarction (MI) is irreversible myocardial cell damage caused by severe and persistent myocardial ischaemia, usually due to the rupture or the erosion of coronary atheroma in the heart, leading to intracoronary thrombosis and occlusion [1]. Alternatively, when coronary atherosclerosis occurs, under adverse haemodynamic effects, blood is redistributed away from the heart muscle supplied by the narrowed coronary arteries [2]. Further heart failure due to MI is a leading cause of mortality [3, 4]. The only way to salvage ischaemic myocardium from MI is the timely perfusion therapy. At present, the main clinical treatment measures for reperfusion in patients with MI include intravenous thrombolysis technology, percutaneous coronary intervention (PCI) and coronary artery bypass grafting (CABG). Revascularisation in patients with MI directly affects prognosis, and timely reperfusion is essential to limit infarct size and salvage ischaemic myocardium. However, myocardial reperfusion can also be accompanied by an exacerbation of myocardial injury, a phenomenon known as myocardial ischaemia–reperfusion injury (MIRI) [4]. Patients with MI usually survive with timely revascularisation, but post‐ischaemic reperfusion injury remains a major driver of heart failure after MI [5, 6, 7]. The benefits and disadvantages of reperfusion therapy for MI have been the focus of debate in the past [8]. However, since 2003, many studies have shown that interventions implemented only during early reperfusion can reduce infarct size, providing clear evidence for reperfusion therapy and its effect on the degree damage caused by MI [9].

The mechanism of MIRI damage involves a variety of molecules and multiple signalling pathways [10], including overproduction of ROS [11], intracellular calcium overload [12], increased catecholamine release and decreased nitric oxide release [13], inflammation [14], rapid normalisation of pH [15], opening of mitochondrial permeability transition pores [16], energy metabolism disorders [17], mitochondrial dysfunction [18], autophagy [19], mitophagy [20, 21], and DNA repair enzyme overactivation [22]. MIRI further worsens cardiac functional outcomes after MI and reduces the potential clinical benefits of medical, interventional and surgical treatments. In clinical trials of coronary ischaemia and reperfusion strategies in developed countries, the rate of recurrence of cardiovascular adverse events in patients with MI after 1 year of standardised treatment was 2%–6% [5, 23], but in practice, the rate was as high as 11% [24]. Therefore, additional myocardial protection is particularly important in parallel with myocardial reperfusion [25]. The prevention and reduction of MIRI have become the focus of current clinical and basic research.

Over the past few decades, mitochondria have been recognised as major regulators of cardiac function and cardiomyocyte viability under physiological and pathological conditions [26, 27]. Structurally, mitochondrial homeostasis is closely regulated by mitochondrial dynamics, including mitochondrial division, mitochondrial fusion and mitophagy [28, 29]. There is substantial evidence that ischaemia–reperfusion (I/R) injury primarily involves a variety of factors, with a focus on the initiation of mitochondrial division [30, 31]. In contrast to mitochondrial division, mitochondrial fusion promotes communication between mitochondria, inhibits the formation of mitochondrial fragments and maintains mitochondrial genome homeostasis [32, 33]. Thus, cells with a well‐coordinated mitochondrial fusion are able to isolate dysfunctional mitochondria and maintain normal cell function. We hope to maintain mitochondrial homeostasis by enhancing mitochondrial fusion to increase the resistance of the heart to myocardial I/R injury.

Molecular hydrogen is the lightest and most abundant element in the Earth's atmosphere. Hydrogen molecules exist in nature in the form of diatomic hydrogen (H_2_), which is considered a natural antioxidant with low reactivity to most biomolecules and potential therapeutic properties. H_2_ has no direct effects on physiological parameters, such as body temperature, blood pressure, pH or PO_2_ [34]. The current application of H_2_ is mainly inhalation, drinking H_2_‐rich water or injecting H_2_ in saline. The first human use of H_2_ was hydreliox, a breathing mixture of H_2_, helium and oxygen, which was used to prevent diver decompression sickness and nitrogen anaesthesia during technical diving at extreme depths [35]. The therapeutic use of H_2_ was first demonstrated in 1975, and hyperbaric H_2_ can significantly regress tumours in mice with cutaneous squamous carcinoma [36]. In 2007, a Japanese scholar reported that inhalation of low concentrations of H_2_ could significantly inhibit brain I/R damage and stroke in rats by buffering oxidative stress [37]. Since then, the biomedical effects of hydrogen, with antioxidant, anti‐inflammatory and antitumour effects as the main research directions, have attracted increasing amount of attention. The therapeutic role of hydrogen has been demonstrated in a variety of diseases, including arteriosclerosis, respiratory diseases, neoplastic diseases and other diseases [38, 39, 40, 41]. However, the current intrinsic therapeutic mechanism of H_2_ has not been fully revealed. Due to its safety and potential efficacy, H_2_ has great potential for clinical application in many diseases.

Mitochondria are called the power source of cells because they produce 90% of the energy that cells need on a daily basis in the form of ATP, a process that relies on oxidative phosphorylation (OXPHOS) and is accompanied by the production of ROS through forward and reverse electron transfers [42]. IR injury markedly increases mitochondrial permeability and dissipates electron and proton gradients. Furthermore, mitochondrial electron transport chain subunits lose their integrity and are degraded under conditions of IR. OXPHOS is markedly diminished in the abnormal mitochondria, leading to bioenergetic insufficiency during MI [43]. H_2_‐mediated improvements in mitochondrial dysfunction are mainly achieved by preventing uncontrolled electron leakage in the electron transport chain. ATP‐sensitive K^+^ (mKATP) channels, located on mitochondria, are important players in energy regulation. In acute MI, H_2_ can modulate the mitochondrial membrane potential and activate mKATP to balance myocardial oxidative CoI levels and mitochondrial DNA (mtDNA) production, thereby attenuating myocardial I/R injury [44]. Therefore, we believe that H_2_ can prevent cell damage by improving mitochondrial function and that the improvement of mitochondrial dysfunction is also expected to alleviate MIRI.

PGC‐1α has been described as a major regulator of mitochondrial biogenesis and function. It is enriched in mitochondria and thermogenic specialised brown adipose tissue along with the peroxisome proliferator‐activated receptor γ (PPARγ) transcription factor. PGC‐1α is known to be specifically expressed in energy‐demanding organs, such as the heart, kidney, brain and skeletal muscle [45, 46, 47, 48, 49]. The PGC‐1α and PPARγ proteins are involved in the regulation of mitochondrial biogenesis, including promoting OXPHOS gene expression in the nucleus and mitochondria and stimulating mtDNA replication, thereby enhancing mitochondrial function and metabolism [50, 51, 52]. PGC‐1α deficiency promotes mitochondrial dysfunction in the progression of various diseases [53].

Therefore, we speculate that H_2_ can alleviate MIRI by improving mitochondrial function by increasing the expression of PGC‐1α during MIRI, providing a new strategy for the treatment of MIRI.

## 2 Materials and Methods

### 2.1 Establishment and Grouping of Isolated Myocardial Ischaemia–Reperfusion (I/R) Injury Model Rats

Male Wistar rats (Beijing Weitong Lihua Laboratory Animal Technology Co. Ltd.) weighing 160–180 g and aged 6 weeks were used to establish the myocardial I/R model. The rats were aged for 1 week in an SPF barrier environment with constant temperature (20°C–25°C), humidity (60%) and light (12:12 h light–dark cycle) and had free access to food and water. The experimental protocol was reviewed and approved by the Animal Experiment Welfare Ethics Committee of Hebei University.

Fasted rats were intraperitoneally injected with heparin (200 U/kg). Fifteen minutes after the administration of heparin, anaesthesia was induced in the rats with 2.5% tribromoethanol (200 mg/kg). After anaesthesia, the abdominal wall was quickly cut along the lower edge of the xiphoid process, the diaphragm was opened, the chest wall was cut along the midline of the thoracic stem, and a thoracotomy was used to stretch the chest to the left and right sides to expose the heart. Without damaging the cardiac aorta, the heart was rapidly removed (within 30 s), the heart was placed in a 4°C saline to remove residual cardiac blood, and the aorta was trimmed and fixed on a Langendorff cardiac perfusion device. Kreb–Henseleit buffer (KHB) containing the following (in mM) at a rate of 8–9.5 mL/min was perfused through the aorta: NaCl, 118.3, KCl, 4.7, CaCL_2_, 2.0, NaHCO_3_, 25.0, KH_2_PO_4_, 1.2, MgSO_4_•7H_2_O, 1.2, glucose, 11.1, pH 7.4 ± 0.5, 37°C ± 0.2°C, 95% O_2_ and 5% CO_2_. Saturated hydrogen‐rich water KHB with 1 g of hydrogen‐rich powder was added to every 100 mL of KHB [the hydrogen‐rich powder was provided by Li Zhilin, a teacher from Hebei University]. Each heart underwent a 10‐min steady heartbeat, 20 min of global ischaemia and 20 min of reperfusion to induce MIRI. The hearts were randomly divided into three groups (*n* = 10): (A) the control group (sham), which was perfused with KHB for 50 min; (B) the ischaemia/reperfusion group (I/R), which was perfused with KHB for 10 min, followed by total myocardial ischaemia for 20 min and reperfusion of KHB for 20 min; and (C) the hydrogen‐rich water I/R group (I/R + H_2_), which was perfused with KHB for 10 min, total myocardial ischaemia for 20 min and saturated hydrogen‐rich water for reperfusion of KHB for 20 min.

### 2.2 Establishment and Grouping of a Hypoxia Reoxygenation Model in H9C2 Cells

Heart‐derived H9C2 cells (HyCyte, China) were cultured with Dulbecco's modified Eagle's medium (DMEM, Gibco, USA) supplemented with 10% foetal bovine serum and 1% penicillin–streptomycin solution in humidified air (5% CO_2_) at 37°C. The medium was changed every 2 days. To establish an in vitro H/R cell model, H9C2 cells were cultured in a 99.9% N_2_‐saturated sugar‐free serum‐free DMEM for 30 min and then maintained in an incubator containing 1% O_2_, 5% CO_2_ and 94% N_2_ for an additional 3 h. After hypoxic incubation, the medium was replaced with DMEM containing 10% foetal bovine serum, and the cells were maintained for 2 h under normoxic conditions (95% air and 5% CO_2_). The experimental cells in this study were divided into five groups: A. control group: H9C2 cells were cultured normally; B. PGC‐1α gene knockdown group (SiRNA): H9C2 cells were transiently transfected for PGC‐1α gene knockdown followed by normal cell culture; C. cell hypoxia/reoxygenation group (HR): normally cultured H9C2 cells were subjected to hypoxia and then reoxygenated with reoxygenation solution; D. cell hypoxia/reoxygenation group under the intervention of hydrogen‐rich water (HR + H_2_): cells were treated as in group C and subjected to a reoxygenation process of hypoxic/saturated hydrogen‐rich water reoxygenation solution for normally cultured H9C2 cells; E. hypoxia/reoxygenation group under hydrogen‐rich water intervention after PGC‐1α gene knockdown (HR + H_2_ + SiRNA): H9C2 cells transiently transfected for PGC‐1α gene knockdown were treated as in group C and subjected to a reoxygenation process of hypoxia/saturated hydrogen‐rich water reoxygenation solution.

### 2.3 Transfection

Transfection with siRNA was used to inhibit PGC‐1α expression in cardiomyocytes. The siRNA against PGC‐1α and the negative control siRNA were purchased from Suzhou GenePharma Biotech Co. Ltd. (Suzhou, China). To transfect the siRNAs into cardiomyocytes, the cells were incubated in OptiMinimal Essential Medium (Invitrogen Life Technologies, Carlsbad, CA, USA) without serum or antibiotics for 24 h. A Lipofectamine 2000 transfection reagent (Invitrogen Life Technologies, Carlsbad, CA, USA) was added to the medium according to the manufacturer's protocol. siRNAs (70 nmol/L/well of siRNA) were subsequently added to the serum‐free medium and incubated with the cells for 72 h. The knockdown efficiency was assessed by Western blotting.

### 2.4 Haematoxylin–Eosin (HE) Staining

Cardiac specimens were fixed with 10% paraformaldehyde, embedded in paraffin, cut into 5‐μm thick sections, placed on slides for HE staining and viewed under an optical microscope.

### 2.5 Transmission Electron Microscopy (TEM)

Mitochondrial ultrastructure in cardiac specimens was evaluated using TEM. In brief, the collected cells were fixed in 2.5% glutaraldehyde overnight at 4°C and fixed in 1% osmium tetroxide for 2 h. After dehydration in a graded ethanol series, the cardiac specimens were infiltrated and embedded in Araldite. Ultrathin sections (60 nm) were cut and stained with 4% uranyl acetate and 0.5% lead citrate. Finally, TEM was used to observe the morphology of the mitochondria and capture images.

### 2.6 qPCR

Total RNA was extracted from the cells using TRIzol reagent (Invitrogen Life Technologies, Carlsbad, CA, USA) and reverse‐transcribed into a total of 1 μL (60 ng/μL) of cDNA using a One‐Step RT‐PCR kit (Vazyme Biotechnology Co. Ltd., Nanjing, China). Gene expression was quantified using an ABI PRISM 7500 Sequence Detection System (Applied Biosystems Life Technologies, Foster City, CA, USA) with SYBR Green (Vazyme Biotechnology Co. Ltd., Nanjing, China). The relative mRNA expression levels were normalised to that of β‐actin using the 2−ΔΔCT method.

### 2.7 Cell Counting Kit‐8 (CCK‐8) Assay

H9C2 cell viability was determined using a CCK‐8 kit according to the manufacturer's instructions. The density of the treated H9C2 cells was adjusted to 1 × 10^5^/mL, and the cells were inoculated in 96‐well plates and cultured overnight at 37°C with 95% O_2_ and 5% CO_2_. CCK‐8 solution (10 μL) was added to each well, and the cells were then cultured for 4 h. An enzyme marker (Thermo, USA) was used to determine the absorbance at 450 nm. Each group was repeated three times.

### 2.8 Lipid ROS Measurement

In brief, H9C2 cells were treated as indicated, and then 50 μL of MC11‐BODIPY581/591 (Glpbio, USA) was added and incubated for 1 h. Excess C11‐BODIPY581/591 was removed by washing the cells twice with PBS. The labelled cells were trypsinised and resuspended in PBS supplemented with 5% FBS. Oxidation of the polyunsaturated butadienyl portion of C11‐BODIPY581/591 resulted in a shift in the fluorescence emission peak from approximately 590 nm to approximately 510 nm proportional to the amount of lipid ROS generated, which was analysed using a flow cytometer (Beckman Coulter, USA). Each experiment was performed in triplicate.

### 2.9 Western Blot

H9C2 cells were lysed with radioimmunoprecipitation assay (RIPA) lysis buffer (Beyotime Institute of Biotechnology, Shanghai, China). A BCA protein assay kit (Beyotime Institute of Biotechnology, China, P0010) was used to measure total protein. The samples (30 μg of protein) were separated by 10% sodium dodecyl sulphate‐polyacrylamide gel electrophoresis (SDS‐PAGE) and subsequently transferred to polyvinylidene difluoride (PVDF) membranes, which were blocked with TBST buffer containing 5% nonfat milk for 1 h at room temperature and incubated with primary antibodies against β‐tubulin (1:20000, Proteintech, China, 66240‐1‐lg), caspase‐3 (1:2000, Abcam, UK, Ab184787), Bax (1:2000, Abcam, UK, Ab32503), Bcl_2_ (1:1000, Abcam, UK, Ab194583), IL‐1β (1:1000, Abcam, UK, Ab254360), TNF‐α (1:1000, Abcam, UK, Ab205587), MFN1 (1:5000, Proteintech, China, 13798–1‐AP), MFN2 (1:5000, Proteintech, China, 12186–1‐AP), OPA1 (1:1000, Proteintech, China, 27733‐1‐AP), PGC‐1α (1:5000, Proteintech, China, 66369‐1‐lg), MFF (1:5000, Proteintech, China, 17090‐1‐AP) and DRP1 (1:2000, Proteintech, China, 12957‐1‐AP) overnight at 4°C. The secondary antibodies (1:10,000, Abways, China, AB0101/AB0102) were diluted in 5% nonfat milk in TBST. The bands were developed using ECL detection reagent. The intensities of the protein bands were measured by ImageJ software.

### 2.10 TUNEL Staining

A terminal deoxynucleotidyl transferase dUTP nick end labelling (TUNEL) assay was used to assess myocardial apoptosis via a TUNEL staining kit (Elabscience Biotechnology Co. Ltd., Wuhan, China) according to the manufacturer's instructions. H9C2 cells were observed under a light microscope with an excitation wavelength of 585–600 nm. TUNEL‐positive nuclei are indicated by blue fluorescence.

### 2.11 Immunofluorescence

H9C2 cells were fixed with 4% paraformaldehyde for 15 min and then were washed with PBS. Triton X‐100 (0.5%) was added to the medium at room temperature for 10 min. The cells were blocked with goat serum for 30 min and then incubated with Cyt‐c primary antibodies (1:500, Proteintech, China, 10993–1‐AP) at 4°C overnight. After the cells were washed with PBS, the excess liquid was removed with absorbent paper, and the cells were incubated with a CoraLite 488‐conjugated goat anti‐rabbit IgG (H + L) secondary antibody (1:500, Proteintech, China, SA00013‐2) at 37°C for 1 h. The nuclei were stained with DAPI (Beyotime Institute of Biotechnology, China) for 5 min. Images were taken under a confocal or fluorescence microscope (Olympus, Japan). The intensities of the target proteins were measured by ImageJ software.

### 2.12 Statistical Analysis

The data in our study are expressed as the mean ± standard error of the mean (SEM) from three independent experiments, each performed in triplicate. Differences among groups were analysed using one‐way analysis of variance (ANOVA) followed by Tukey's post hoc test. Values of *p* < 0.05 indicated that the differences were statistically significant. The statistical data were analysed with GraphPad Prism 8 software.

## 3 Results

### 3.1 H_2_ Reduces Myocardial I/R Damage

**FIGURE 1:**
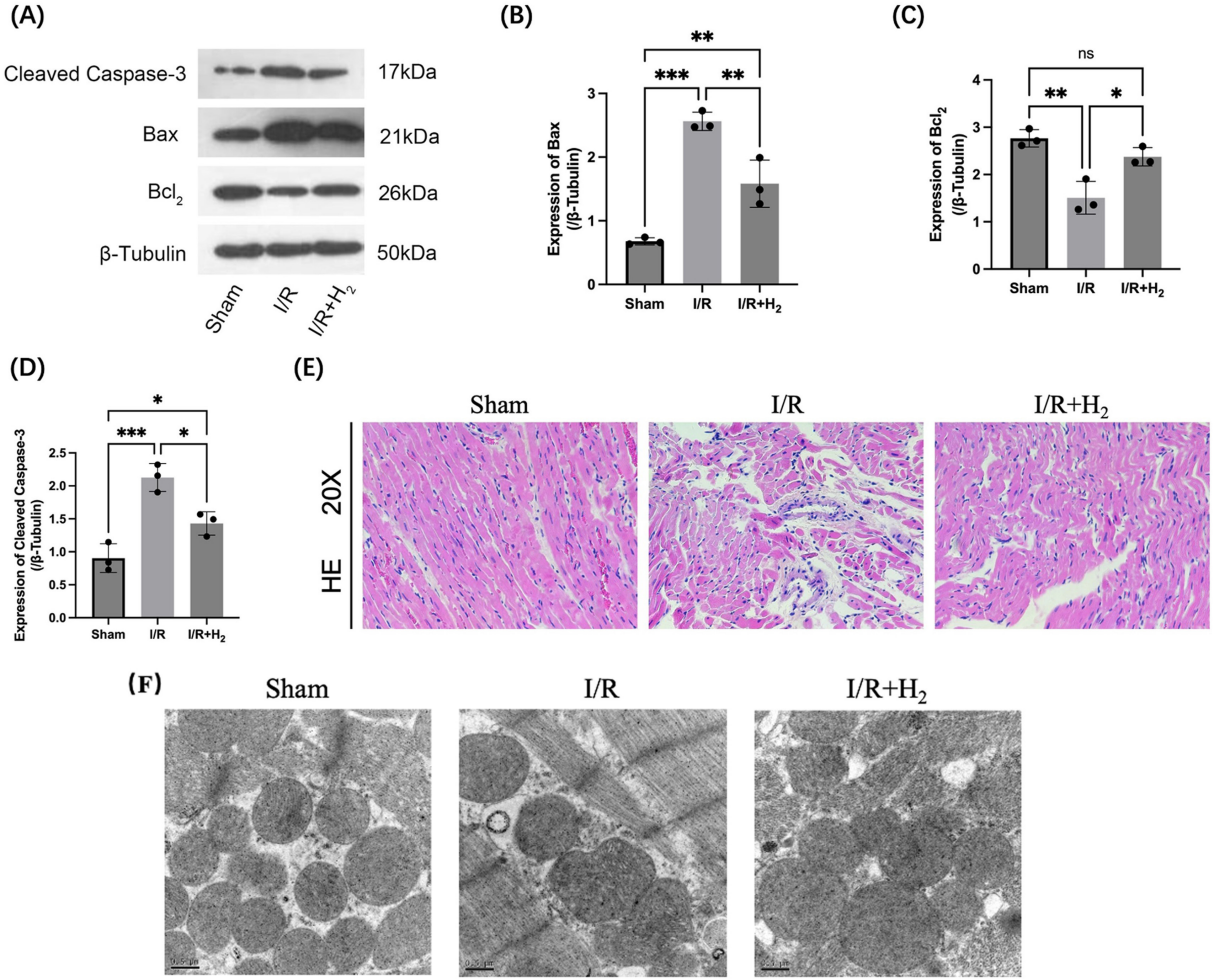
H_2_ may reduce myocardial I/R damage. A–D, Western blotting was used to detect the expression levels of the apoptosis markers Bax, cleaved caspase‐3 and Bcl_2_ after myocardial I/R injury. E, HE staining was used to observe the effect of I/R damage on the myocardial tissue structure. F, Typical image of the mitochondrial structure of myocardial tissue under TEM. *n* = 10; the data are expressed as $\bar{x}\pm s$; ns is not statistically significant, *p < 0.05, **p < 0.01, ***p < 0.001.

We first verified whether H_2_ exerts a beneficial effect on myocardial I/R injury. The expression levels of apoptosis markers in myocardial tissue were detected by Western blot. Compared with those in the sham operation group, the expression levels of Bax and cleaved caspase‐3, which promote apoptosis, increased in myocardial tissue, and the expression of Bcl_2_, a marker that inhibits apoptosis, decreased in the I/R group, suggesting that apoptosis occurred during I/R in myocardial tissue. Compared with those in the I/R group, the expression levels of Bax and cleaved caspase‐3, which are markers of apoptosis, decreased in the I/R + H_2_ group, and the level of Bcl_2_, a marker that inhibits apoptosis, increased, suggesting that the apoptosis that occurred during I/R in myocardial tissue could be reversed by H_2_ (Figure 1A–D). HE staining was used to assess the morphology of the rat cardiomyocytes, and the images (Figure 1E) revealed that the cardiomyocytes in the sham group were arranged in a regular manner, with obvious nuclei and no marked inflammatory cell infiltration. The myocardial tissue of the I/R group showed extensive necrosis of myocardial cells, disordered arrangement of myocardial fibres and a large number of inflammatory cell infiltrates. Compared with those in the I/R group, the cardiomyocytes in the I/R + H_2_ group were more orderly, and the degree and extent of cell necrosis were significantly lower. Morphological changes in mitochondria were observed by TEM (Figure 1F), and I/R‐induced ultrastructural changes in myocardial tissue included poor mitochondrial outer membrane integrity, mitochondrial relaxation and swelling, and decreased intermitochondrial interstitial distance. Compared with those in the I/R group, the mitochondrial changes in the I/R + H_2_ group were significantly alleviated. The above results showed that H_2_ treatment significantly reduced I/R‐induced myocardial tissue damage in rats.

### 3.2 Deletion of PGC‐1α Attenuates the Protective Effect of H_2_ on Ischaemia–Reperfusion‐Induced Heart Injury

**FIGURE 2:**
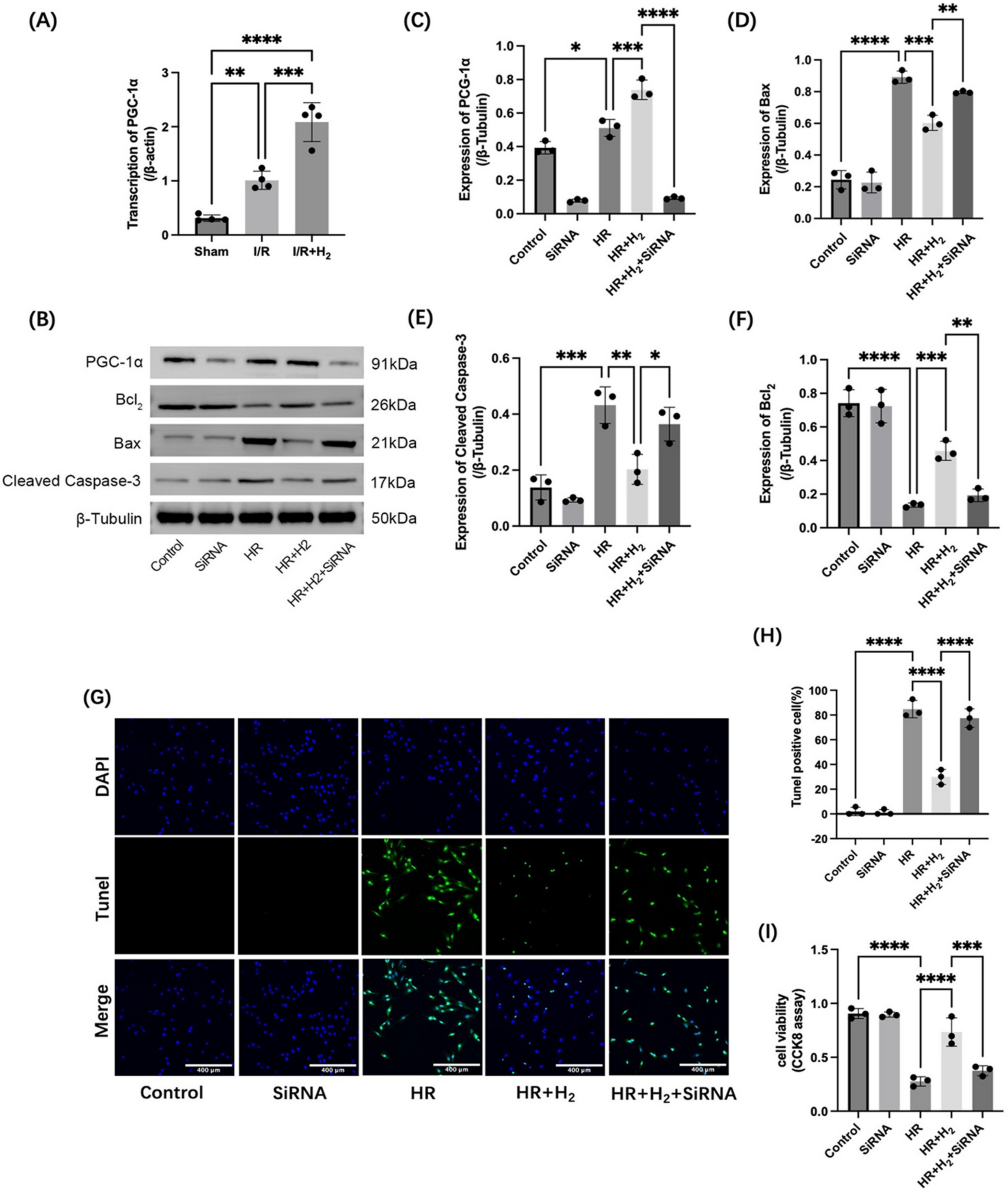
Deletion of PGC‐1α weakens the protective effect of H_2_ on I/R injury to the heart. A, qPCR was used to detect PGC‐1α transcription in the rat myocardial tissue after I/R injury. B–F, Western blotting was used to detect the expression levels of PGC‐1α and the apoptosis markers Bax, cleaved caspase‐3 and Bcl_2_. G, H, TUNEL was used to detect the extent of H9C2 myocardial cell apoptosis. I, H9C2 cell viability was determined by a CCK‐8 assay. A: *n* = 4, B–I: *n* = 3; the data are expressed as $\bar{x}\pm s$; *p < 0. 05, **p < 0.01, ***p < 0.001, ****p < 0.0001.

The expression levels of PGC‐1α in the I/R and I/R + H_2_ groups were detected by qPCR and compared with that in the sham group, the expression of PGC‐1α in the myocardial tissue was stimulated by I/R injury, and the PGC‐1α level further increased with the use of H_2_ (Figure 2A). Western blot analysis revealed that H/R damage significantly increased the PGC‐1α expression in H9C2 cells compared to that in the control group, and H_2_ further enhanced this alteration, suggesting that hydrogen‐rich water promotes HR‐mediated PGC‐1α upregulation (Figure 2B,C). To verify whether PGC‐1α is required for H_2_‐mediated cardioprotection, we performed transient transfection to knock down the PGC‐1α gene in H9C2 rat cardiomyocytes and compared the cell damage parameters after HR injury and H_2_ treatment with those after HR injury. The mortality rate of H9C2 cardiomyocytes was observed using the TUNEL assay, and as shown in the images (Figure 2G,H), the cell mortality rate was greater than 80% after HR injury and significantly lower (less than 30%) after H_2_ treatment. Deletion of PGC‐1α reactivated cell death despite H_2_ treatment. Cell viability was determined by the CCK‐8 method, and H9C2 cell viability was significantly reduced due to HR damage compared to that of the control group, which returned to near‐normal levels after H_2_ treatment, though the protective effect of H_2_ was abrogated in PGC‐1α‐deficient H9C2 cardiomyocytes (Figure 2I). Similar results were obtained by observing apoptosis by Western blot (Figure 2B,D–F). HR injury increased the expression levels of the pro‐apoptotic markers Bax and cleaved caspase‐3, while H_2_ treatment prevented the activation of Bax and caspase‐3 in a PGC‐1α‐dependent manner. HR damage also reduced the expression of the inhibitory apoptotic marker Bcl_2_, which activates Bcl_2_ in a PGC‐1α‐dependent manner. Both in vivo I/R and in vitro HR experiments showed that H_2_ could protect the reperfused heart from I/R damage by increasing the expression of PGC‐1α.

### 3.3 H_2_ Ameliorates the Cardiomyocyte Inflammatory Response and Oxidative Stress Under HR Injury by Acting on PGC‐1α

**FIGURE 3:**
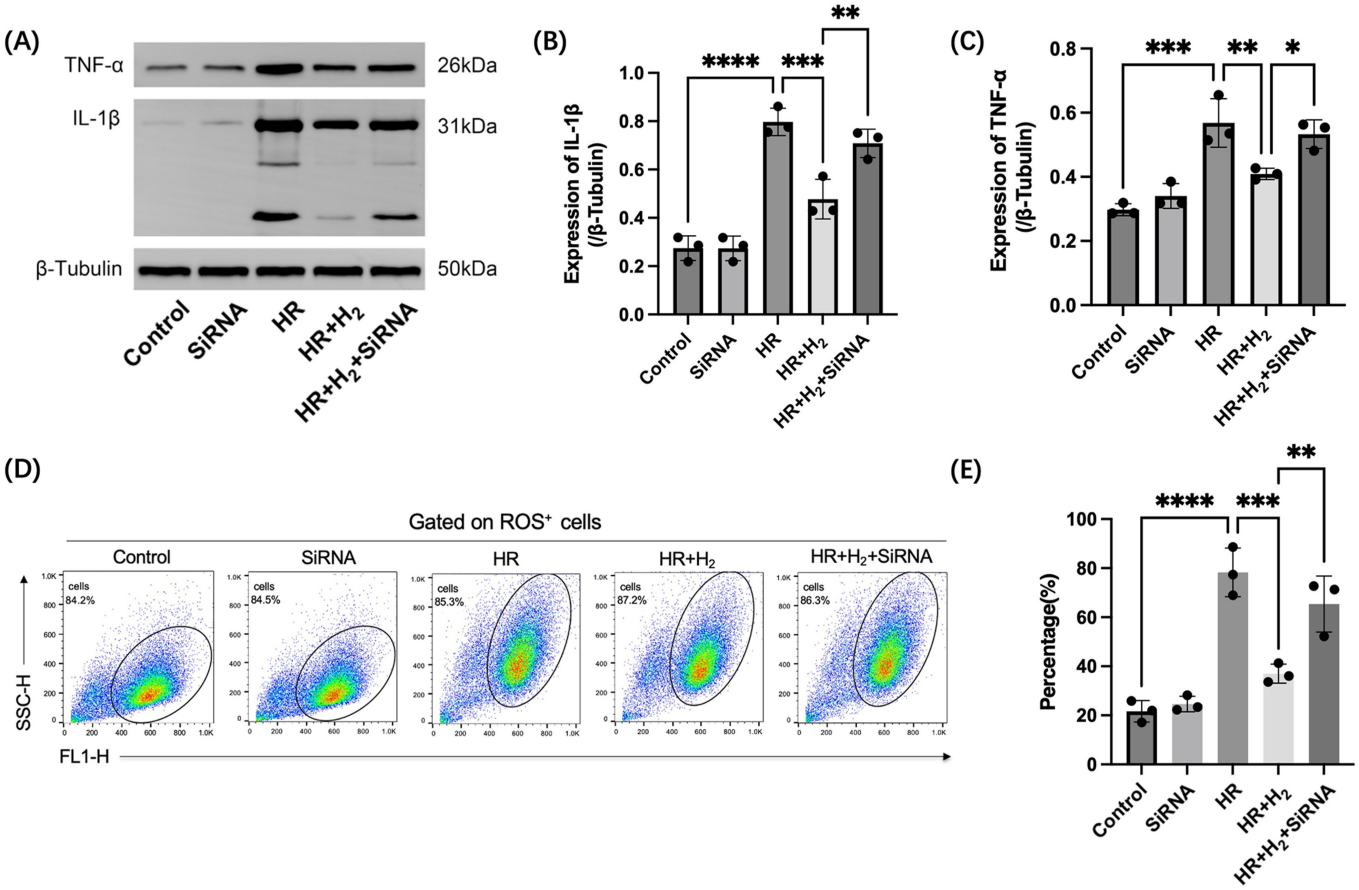
H_2_ ameliorates the cardiomyocyte inflammatory response and oxidative stress under HR injury by acting on PGC‐1α. A–C, Western blotting was used to detect the expression levels of IL‐1β and TNF‐α in H9C2 rat cardiomyocytes. D, E, Flow cytometry was used to measure the level of ROS in H9C2 cells. *n* = 3; the data are expressed as $\bar{x}\pm s$; *p < 0.05, **p < 0.01, ***p < 0.001, ****p < 0.0001.

Western blot analysis revealed that the levels of inflammatory factors, such as TNF‐α and interleukin‐1β (IL‐1β), in H9C2 cells were significantly increased after HR injury, while H_2_ treatment effectively inhibited the expression of TNF‐α and IL‐1β, which was achieved by increasing the expression of PGC‐1α; however, the deletion of PGC‐1α inhibited the therapeutic effect of H_2_ (Figure 3A–C). In addition, a ROS assay evaluated by flow cytometry demonstrated that HR damage caused oxidative stress in cells, which was reversed to near‐normal levels after H_2_ treatment. Moreover, treatment with H_2_ failed to attenuate ROS production when PGC‐1α was deficient, suggesting that H_2_ treatment could attenuate HR‐induced cellular oxidative damage and that the effect of H_2_ on PGC‐1α‐deficient cardiomyocytes was negated (Figure 3D,E).

### 3.4 H_2_ Exerts Mitochondrial Protective Effects in a PGC‐1α‐Dependent Manner

**FIGURE 4:**
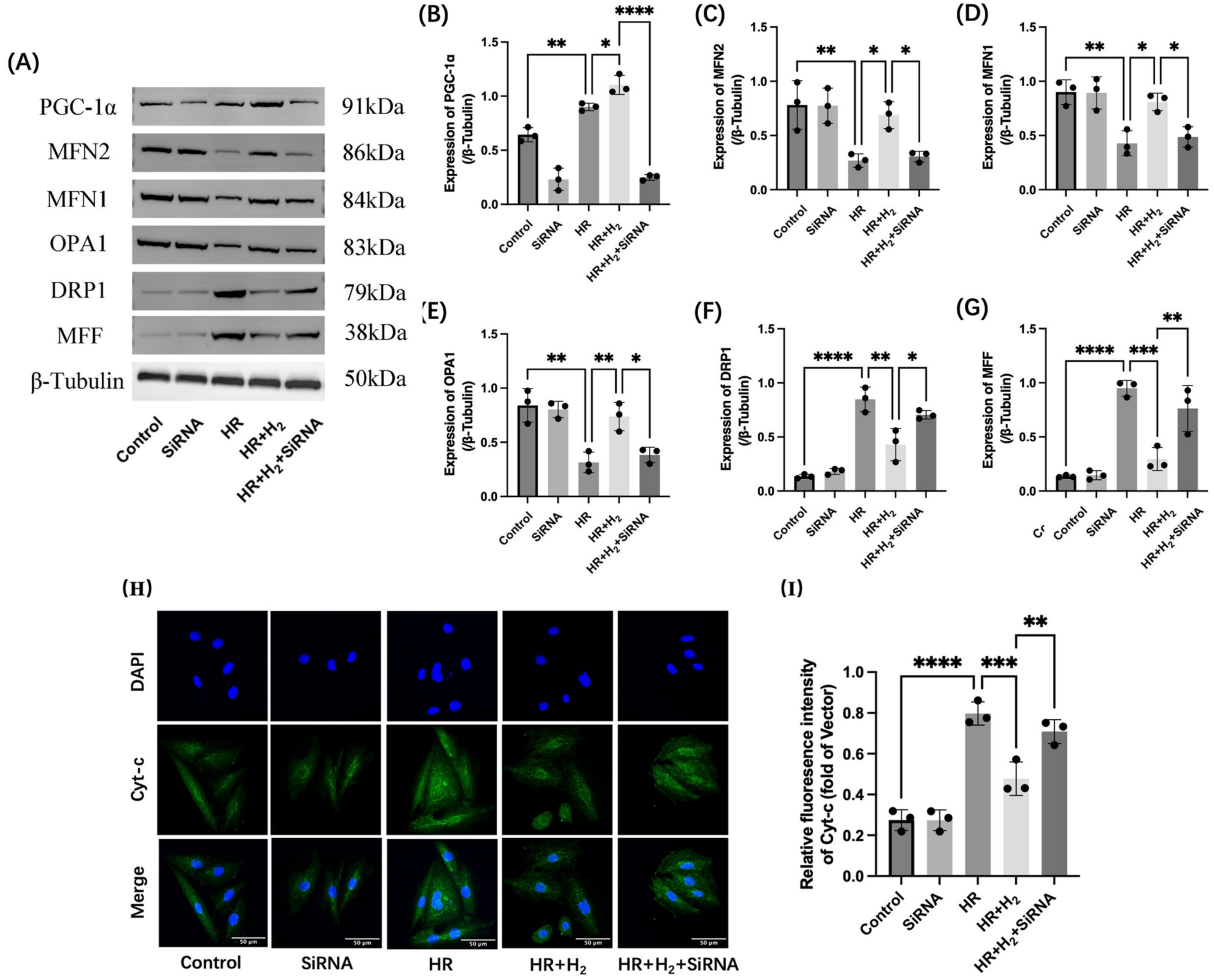
H_2_ protects mitochondrial function under HR injury conditions in H9C2 rat cardiomyocytes, and this effect is dependent on the normal expression of PGC‐1α. A–G, Western blotting was used to detect the expression levels of PGC‐1α, MFN1, MFN2, DRP1, OPA1 and MFF in H9C2 cells. H, I, Immunofluorescence was used to detect the expression level of Cyt‐c in H9C2 cells. *n* = 3; the data are expressed as $\bar{x}\pm s$; *p < 0.05, **p < 0.01, ***p < 0.001, ****p < 0.0001.

Because PGC‐1α is an important regulator of mitochondrial biogenesis and function and mitochondrial damage is an important factor in MIRI, we performed experiments to analyse the combined role of H_2_ and PGC‐1α in mitochondrial apoptosis. Mitochondrial apoptosis is characterised by the release of the pro‐apoptotic factor cytochrome C (Cyt‐c) from the mitochondria into the cytoplasm. Immunofluorescence (Figure 4H,I) revealed that more Cyt‐c appeared in the cytoplasm/nucleus after H9C2 cells were subjected to HR damage, though H_2_ treatment effectively inhibited the release of Cyt‐c, while the deletion of the PGC‐1α gene eliminated the beneficial effects of H_2_. Subsequently, the expression levels of mitochondrial fission– and mitochondrial fusion–related proteins were detected using Western blotting. As shown in Figure 4A–J, the expression levels of pro‐fission proteins (e.g., DRP1 and MFF) were significantly upregulated in HR‐treated H9C2 cells compared to control H9C2 cells, while the expression levels of pro‐fusion factors (e.g., MFN1, MFN2 and OPA1) were significantly downregulated in response to HR injury. H_2_ treatment increased the expression of the pro‐fusion protein while decreasing the expression of the pro‐fission protein. Deletion of PGC‐1α abolished the regulatory effect of H_2_ water on mitochondrial fusion and fission. These results confirmed that H_2_ plays an important role in the survival of H9C2 cells under HR injury by promoting mitochondrial fusion and inhibiting mitochondrial division through PGC‐1α.

## 4 Discussion

Hydrogen is therapeutic for MIRI. As an important indicator of tissue damage, apoptosis was induced to establish an ex vivo myocardial I/R injury model in rats, and the expression levels of myocardial apoptosis markers were detected by Western blotting. I/R damage significantly increased the expression levels of pro‐apoptotic markers (Bax and cleaved caspase‐3) and decreased the expression of an inhibitory apoptotic marker (Bcl_2_). H_2_ effectively alleviated apoptosis during myocardial tissue I/R injury. Rat cardiomyocyte morphology was observed using HE staining to assess the extent of tissue damage. Cardiomyocytes in the sham group were regularly arranged, with obvious nuclei and no inflammatory cell infiltration. Myocardial tissue after I/R injury exhibited signs of extensive myocardial cell necrosis, myocardial fibre arrangement and massive inflammatory cell infiltration. After H_2_ treatment, the arrangement of cardiomyocytes was more orderly, and the degree and extent of tissue necrosis were significantly reduced. Morphological changes in mitochondria were observed by TEM, and the I/R‐induced mitochondrial ultrastructural changes in the myocardial tissue included poor mitochondrial membrane integrity, mitochondrial relaxation and swelling, and decreased mitochondrial intercrest distance. The mitochondrial changes in the group were significantly alleviated after H_2_ treatment. The above results show that the use of H_2_ significantly reduces the I/R‐induced myocardial tissue damage in rats and that H_2_ is a therapeutic agent for MIRI.

We performed qPCR to detect the expression of PGC‐1α in the rat myocardial tissue after I/R injury with and without subsequent H_2_ treatment. PGC‐1α expression was increased by I/R injury, and its expression was further increased by H_2_ application. To verify whether the increase in PGC‐1α expression is related to the therapeutic effect of H_2_ on MIRI, the PGC‐1α gene in rat H9C2 cardiomyocytes was knocked down by transient transfection, and an HR model of rat H9C2 cardiomyocytes was established. CCK8 analysis showed that H_2_ could improve the decrease in cell viability caused by HR injury, but the therapeutic effect of H_2_ was not significant for cells with PGC‐1α gene knockdown. Western blotting was used to detect the expression of apoptosis markers, and it was found that the application of H_2_ could alleviate apoptosis caused by HR damage in cells, though the effect of H_2_ treatment depended on the normal expression of PGC‐1α. The apoptosis rate observed by TUNEL staining also confirmed the above conclusions. These results demonstrated that the therapeutic effect of H_2_ on I/R damage was achieved by increasing the expression of PGC‐1α.

Inflammation is an integral process of the body that defends against adverse environmental factors by strengthening the balance of defences in the body, tissue integrity, and organ function and structure. However, persistent inflammation is considered the primary cause of almost all diseases and underlies a wide range of physiological and pathological processes [54]. The activation and upregulation of PGC‐1α through genetic or pharmacological manipulation can inhibit inflammation and exert protective effects in different pathological models [54, 55, 56, 57]. In this study, Western blot analysis revealed that the levels of IL‐1β and TNF‐α in cells increased significantly after HR injury, while H_2_ treatment effectively reduced the expression of these two inflammatory factors. However, for H9C2 rat cardiomyocytes with PGC‐1α gene knockdown, the effect of H_2_ treatment was significantly weakened after HR injury. The results showed that the inflammation level of cells increased significantly when HR injury occurred, and H_2_ effectively improved the inflammatory response during HR injury, though the efficacy of H_2_ treatment depended on the normal expression of PGC‐1α.

Oxidative stress refers to an imbalance between the oxidative system and antioxidant defences, which is caused by an overproduction of ROS or reactive nitrogen and ultimately leads to damage to DNA, proteins and cells [58]. PGC‐1α affects the expression of the OXPHOS gene and oxidative stress response genes in human, mouse and bovine endothelial cells [59]. Preceding investigations have demonstrated that the SIRT1‐PGC‐1α–SIRT3 signalling mechanism adopted by melatonin indirectly activates the downstream signalling pathways that not only induces mitochondrial biogenesis but also prevents mitochondrial dysfunction in rat heart against ISO‐induced oxidative stress [60]. Oxidative stress is an important intrinsic mechanism of MIRI, and flow cytometry was used to explore the alteration of ROS in the HR model of H9C2 cardiomyocytes. Compared with those in the control group, the levels of ROS significantly increased after HR injury, suggesting that HR damage caused oxidative stress in cells. After H_2_ treatment, the level of ROS decreased, indicating that H_2_ could effectively alleviate oxidative stress caused by HR injury. However, H_2_ failed to achieve the same therapeutic effect in the HR damage model of cells after knockdown of the PGC‐1α gene. H_2_ has been demonstrated to have a therapeutic effect on oxidative stress after HR injury in cells, and this therapeutic effect needs to be achieved by relying on PGC‐1α.

Cyt‐c has life‐sustaining and cell death–related functions, depending on its subcellular localisation. Within mitochondria, Cyt‐c acts as a single electron carrier as part of the electron transport chain. When Cyt‐c is released into the cytoplasm after cell injury, it triggers the assembly of apoptotic bodies, causing intrinsic apoptosis. Immunofluorescence was used to compare the release of Cyt‐c from H9C2 rat cardiomyocytes before and after HR injury, and it was found that the release of Cyt‐c was significantly increased after HR, while the release amount was significantly decreased by H_2_ treatment, and there was no obvious effect of H_2_ treatment on cells with PGC‐1α gene knockdown. These results inferred that H_2_ treatment can improve apoptosis after HR injury in cells, and this therapeutic effect is achieved in a PGC‐1α‐dependent manner. As part of an energy‐intensive organ, cardiomyocytes are rich in mitochondria. The maintenance of the cellular mitochondrial function and adaptation to changing energy demands are regulated by the remodelling of mitochondrial structure, which is primarily controlled by fission/fusion, mitochondrial biogenesis and mitochondrial phagocytosis [61]. Normal mitochondrial fission promotes mitochondrial redistribution, while fusion maintains a healthy mitochondrial network. Abnormal mitochondrial fission is thought to be a prerequisite for the initiation of intrinsic apoptosis in cardiomyocytes during the I/R injury phase by inducing damage to mtDNA copy/transcription, interrupting mitochondrial bioenergetics and triggering mitochondrial dysfunction and cell death pathways. The mitochondrial protein MFN1 leads to the mitochondrial outer membrane fusion, MFN2 and OPA1 mediate the mitochondrial inner membrane fusion, and DPR1 and MFF mediate mitochondrial division. In the present study, the expression levels of mitochondrial fission– and mitochondrial fusion–related proteins were detected using Western blotting. HR damage reduced the expression levels of the pro‐fusion proteins MFN1/2 and OPA1 and increased the expression levels of the pro‐fission proteins DPR1 and MFF, and H_2_ activated mitochondrial fusion and inhibited mitochondrial division in a PGC‐1α‐dependent manner, thereby alleviating cellular HR damage.

In summary, we have experimentally demonstrated that H_2_ can treat MIRI, alleviate apoptosis, oxidative stress and inflammatory responses, regulate mitochondrial fission/fusion and protect mitochondrial function in a PGC‐1α‐dependent manner. In this study, evidence was provided not only to explain the important role of PGC‐1α‐associated mitochondrial protection in cardiac I/R injury but also to explore the role of PGC‐1α‐associated myocardial protection in cardiac I/R injury under the influence of H_2_.

Finally, research on the preventive and therapeutic effects of H_2_ on many diseases is still in its infancy. Further in‐depth studies are needed due to the significant differences in experimental strategies between animal/cultured cell efficacy testing and clinical trials. While all the findings suggest that H_2_ is a novel and promising antioxidant, its potential must be further validated in animal experiments and in the clinic.

## 5 Conclusion

The main conclusions of this study are as follows: the application of H_2_ can reduce myocardial I/R injury, the deletion of PGC‐1α weakened the protective effect of H_2_ on I/R heart, H_2_ ameliorates myocardial cell inflammatory response and oxidative stress under HR injury by acting on PGC‐1α, H_2_ can exert mitochondrial protection function and this effect is absolved by PGC‐1α deletion under the condition of HR injury. The above conclusions suggest that hydrogen protects mitochondrial function by increasing the expression of PGC‐1α and improves MIRI.

## Author Contributions

**Yue Zuo:** data curation (equal), formal analysis (equal), investigation (equal), writing – original draft (equal). **Jiawei Wang:** data curation (equal), formal analysis (equal), investigation (equal), methodology (equal), project administration (equal). **Zhexuan Gong:** data curation (equal), investigation (equal). **Yulong Wang:** investigation (equal), methodology (equal). **Qiang Wang:** data curation (equal), investigation (equal). **Xueyang Yang:** investigation (equal). **Fulin Liu:** conceptualization (equal), funding acquisition (equal), project administration (equal), writing – review and editing (equal). **Tongtong Liu:** formal analysis (equal), project administration (equal), writing – review and editing (equal).

## Ethics Statement

The present study was performed at the Central Laboratory of the Affiliated Hospital of Hebei University. All animal experiments were approved by the Animal Ethical and Welfare Committee of Hebei University (Baoding, China, approval no. IACUC‐2022007SR) and were performed in accordance with the Guidelines for the Care and Use of Laboratory Animals of Hebei University.

## Consent

The authors have nothing to report.

## Conflicts of Interest

The authors declare no conflicts of interest.

## Data Availability

All raw data images and materials that are presented in this paper will be made available for reviewers when necessary.
